# Contribution of local regeneration of glucocorticoids to tissue steroid pools

**DOI:** 10.1530/JOE-23-0034

**Published:** 2023-07-28

**Authors:** S Khan, D E W Livingstone, A Zielinska, C L Doig, D F Cobice, C L Esteves, J T Y Man, N Z M Homer, J R Seckl, C L MacKay, S P Webster, G G Lavery, K E Chapman, B R Walker, R Andrew

**Affiliations:** 1 Centre for Cardiovascular Science, Queen’s Medical Research Institute, University of Edinburgh, Edinburgh, UK; 2 Centre for Discovery Brain Science, University of Edinburgh, Hugh Robson Building, Edinburgh, UK; 3 College of Medical and Dental Sciences, University of Birmingham, Birmingham, UK; 4 Department of Biosciences, School of Science & Technology, Nottingham Trent University, Nottingham, UK; 5 Mass Spectrometry Core Laboratory, Edinburgh Clinical Research Facility, Queen’s Medical Research Institute, University of Edinburgh, Edinburgh, UK; 6 SIRCAMS, School of Chemistry, University of Edinburgh, Joseph Black Building, King's Buildings, Edinburgh, UK; 7 Clinical & Translational Research Institute, Newcastle University, International Centre for Life, Central Parkway, Newcastle upon Tyne, UK

**Keywords:** 11β-hydroxysteroid dehydrogenase 1, hexose-6-phosphate dehydrogenase, glucocorticoid, liver, adipose tissue, brain

## Abstract

11β-Hydroxysteroid dehydrogenase 1 (11βHSD1) is a drug target to attenuate adverse effects of chronic glucocorticoid excess. It catalyses intracellular regeneration of active glucocorticoids in tissues including brain, liver and adipose tissue (coupled to hexose-6-phosphate dehydrogenase, H6PDH). 11βHSD1 activity in individual tissues is thought to contribute significantly to glucocorticoid levels at those sites, but its local contribution vs glucocorticoid delivery via the circulation is unknown. Here, we hypothesised that hepatic 11βHSD1 would contribute significantly to the circulating pool. This was studied in mice with Cre-mediated disruption of *Hsd11b1* in liver (*Alac*-Cre) vs adipose tissue (*aP2*-Cre) or whole-body disruption of *H6pdh*. Regeneration of [9,12,12-^2^H_3_]-cortisol (d3F) from [9,12,12-^2^H_3_]-cortisone (d3E), measuring 11βHSD1 reductase activity was assessed at steady state following infusion of [9,11,12,12-^2^H_4_]-cortisol (d4F) in male mice. Concentrations of steroids in plasma and amounts in liver, adipose tissue and brain were measured using mass spectrometry interfaced with matrix-assisted laser desorption ionisation or liquid chromatography. Amounts of d3F were higher in liver, compared with brain and adipose tissue. Rates of appearance of d3F were ~6-fold slower in *H6pdh^−/−^* mice, showing the importance for whole-body 11βHSD1 reductase activity. Disruption of liver 11βHSD1 reduced the amounts of d3F in liver (by ~36%), without changes elsewhere. In contrast disruption of 11βHSD1 in adipose tissue reduced rates of appearance of circulating d3F (by ~67%) and also reduced regenerated of d3F in liver and brain (both by ~30%). Thus, the contribution of hepatic 11βHSD1 to circulating glucocorticoid levels and amounts in other tissues is less than that of adipose tissue.

## Introduction

11β-Hydroxysteroid dehydrogenase 1 (11βHSD1) generates active 11-hydroxy glucocorticoids (cortisol (human), corticosterone (rodent and human)) from intrinsically inert 11-keto steroids (cortisone, 11-dehydrocorticosterone (11DHC), respectively) (Anderson & Walker 2013, Chapman *et al.* 2013). 11βHSD1 acts in conjunction with hexose-6-phosphate dehydrogenase (H6PDH), which supplies its NADPH co-factor (Lavery *et al.* 2006). Chronic glucocorticoid excess causes a spectrum of adverse effects including type 2 diabetes, hypertension, visceral obesity, myopathy, mood disturbances and cognitive deficits, and 11βHSD1 has therefore emerged as a drug target to reduce active glucocorticoid levels (Hughes *et al.* 2008) in a tissue-specific manner. This can be achieved by inhibiting 11βHSD1 directly or through restricting co-factor availability. Some drug candidates have reached clinical trials (Rosenstock *et al.* 2010, Feig *et al.* 2011, Shah *et al.* 2011, Heise *et al.* 2014).

Initial trials investigated the potential of 11βHSD1 inhibitors to improve glycaemic control in patients with type 2 diabetes mellitus, based on pre-clinical studies showing an improved metabolic phenotype in mice with a targeted disruption of the *Hsd11b1* gene (Kotelevtsev *et al.* 1997, Morton *et al.* 2004). Short-term experimental studies of inhibitors in rodents (Hermanowski-Vosatka *et al.* 2005, Liu *et al.* 2011) and humans (Andrews *et al.* 2003, Sandeep *et al.* 2005) supported this concept but did not achieve better endpoints than current therapies (Rosenstock *et al.* 2010, Feig *et al.* 2011, Shah *et al.* 2011, Heise *et al.* 2014). Brain penetrant 11βHSD1 inhibitors have been evaluated as potential therapies for Alzheimer’s disease and other age-related cognitive impairments (Yau *et al.* 2001, 2007, 2011, 2015, Webster *et al.* 2007, Katz *et al.* 2013, Sooy *et al.* 2015), with candidates (Webster *et al.* 2016) currently in phase II clinical trials (e.g., NCT05657691). This therapeutic concept has evolved from preclinical studies demonstrating beneficial effects upon cognition in aged animals following reductions in whole-body levels of glucocorticoids in mice (Yau *et al.* 2001, 2007, 2011, 2015). This can be achieved bluntly by removing the adrenal glands which are the major source of endogenous glucocorticoids (Montaron *et al.* 2006), with the risk of Addisonian crisis, but also more subtly by reducing 11βHSD1 activity through lifelong genetic disruption (Yau *et al.* 2001, 2007, 2011) or with short-term pharmacological inhibition of 11βHSD1 activity (Sooy *et al.* 2010, 2015, Webster *et al.* 2016). The potential of 11βHSD1 inhibitors to improve wound healing is under clinical translation (Ajjan *et al.* 2022) and preclinical studies highlight the opportunities for 11βHSD1 inhibitors to enhance tissue repair after myocardial infarction (Mylonas *et al.* 2017). Thus, there are many applications of the drug class, each requiring assessment of changes in amounts of active glucocorticoids (and ratios vs inactive steroids) in specific tissue sites.

Recent advances in pharmacodynamic monitoring through mass spectrometry (MS) have allowed tracing of glucocorticoids *in situ* and showed suppression of the ratio of active/inert (cortisol/cortisone and corticosterone/11-DHC) in response to 11βHSD1 inhibition in brain regions (Cobice *et al.* 2017). However, it remains unclear the extent to which *11βHSD1* in individual tissues contributes to the circulating pool of active steroids and how changes in glucocorticoid regeneration in one tissue can influence active steroid levels in another, if at all. In humans, contributions of 11βHSD1 within different tissues to whole-body regeneration of active glucocorticoids have been explored by tracer kinetics in conjunction with arteriovenous sampling and biopsy. The reductive activity of 11βHSD1 has been tracked using a dilution of the administered tracer [9,11,12,12-^2^H_4_]-cortisol (d4F) by [9,12,12-^2^H_3_]-cortisol (d3F), which reflects the steroid regenerated by 11β-reduction, via the intermediate [9,12,12-^2^H]_3_-cortisone (d3E) (Andrew *et al.* 2002). This approach has the advantage over the use of the crude ratio of endogenous steroids where source pools of cortisol cannot be distinguished. Furthermore, the commonly used ratio of endogenous steroids reflects a balance of reductase and dehydrogenase activities, where dehydrogenation can be catalysed by both 11βHSD1 and the type 2 isozyme, 11βHSD2 (Hughes *et al.* 2012, Anderson *et al.* 2021). Using tracer kinetics, Cobice *et al.* (Cobice *et al.* 2017) showed the unique role of 11βHSD1 to form d3F using global 11βHSD1 knockout mice, who were unable to recycle active glucocorticoid from the d4F tracer. Additionally, H6PDH has received attention in determining the reaction direction of 11βHSD1, but its contribution and relative tissue contribution have not been quantified through tracer kinetics.

The measurement across the liver by arterio-venous sampling of production rates of d3F regenerated by 11βHSD1 (Andrew *et al.* 2005, Basu *et al.* 2006, 2009) supports the view that, in humans, the majority of circulating cortisol arising from 11βHSD1-mediated glucocorticoid regeneration derives from the hepatic enzyme. Measurable production of cortisol generated by 11βHSD1, albeit at much slower rates, has also been quantified across adipose tissue and skeletal muscle (Hughes *et al.* 2013) but not across the human brain (Kilgour *et al.* 2015) or heart (Iqbal *et al.* 2014). These dynamic findings are consistent with the higher expression of 11βHSD1 in liver than in other tissues and strongly suggest that hepatic 11βHSD1 exerts the biggest (non-adrenal) influence on the active and inactive glucocorticoids substrate and product in the circulation. Indeed transgenic over-expression of 11βHSD1 in the liver can correct the hypothalamic–pituitary–adrenal axis (HPA) phenotype of global 11βHSD1 deficiency (Paterson *et al.* 2007). Here we tested the hypothesis that reduced hepatic 11βHSD1 reductase activity will decrease the proportion of the circulating pool of cortisol derived through reduction of inert keto-steroids and also reduce exposure of the brain and adipose tissue to regenerated active glucocorticoid. This is of relevance in understanding the consequences of tissue-specific up/down regulation of the enzyme and also design of the pharmacodynamic profiles of potential inhibitors.

## Materials and methods

### Materials

[9,11,12,12-^2^H]_4_-Cortisol (d4F), [9,12,12-^2^H]_3_-cortisol (d3F) and corticosterone were from Cambridge Isotopes (Tewksbury, MA, USA). For LC-MS/MS [9,11,12,12-^2^H]_4_-cortisol (certified reference material) was from Cerilliant (Round Rock, TX, USA), [2,2,4,6,6,9,12,12-^2^H]_8_-cortisone (d8E) from Sigma Aldrich (Poole, Dorset, UK) and [2,2,4,6,6,7,21,21-^2^H]_8_-corticosterone (d8B) from CK Isotopes (Unthank, Leicestershire, UK). Solvents (methanol, acetonitrile and water) were glass-distilled HPLC and LC-MS grades (Fisher Scientific, Leicestershire, UK). α-Cyano-4-hydroxy cinnamic acid (CHCA), trifluoracetic acid, ammonium fluoride (NH_4_F) and all other chemicals were from Sigma-Aldrich unless stated. Room temperature (RT) was 18–21°C.

### Animal models and husbandry

Male mice (*mus musculus*) were studied aged 9–12 weeks, congenic on a C57Bl/6J genetic background; *Hsd11b1^f/f^* mice, with *LoxP* sites flanking exon 3 of the *Hsd11b1* gene, were generated by Taconic Artemis (Cologne, Germany) (Verma *et al.* 2018). *Hsd11b1^LKO^* (LKO) mice, with hepatocyte 11βHSD1 deficiency, previously described with 94–100% knockdown (Zou *et al.* 2018), were generated by crossing Alb-Cre transgenic mice with *Hsd11b1^f/f^* mice. *Hsd11b1^AKO^* (AKO) mice, with adipocyte 11βHSD1 deficiency, were generated by crossing *aP2-Cre* transgenic mice (He *et al.* 2003) with *Hsd11b1^f/f^* mice. Experimental *Hsd11b1^LKO^* or *Hsd11b1^AKO^* mice were the offspring of male *Hsd11b1^LKO^* mice or male *Hsd11b1^AKO^* mice, respectively, each bred with female *Hsd11b1^f/f^* mice. Controls were *Hsd11b1^f/f^* littermates. Knockdown of *Hsd11b1* was achieved in adipose tissue in the AKO colony compared to control littermates and is demonstrated in Supplementary Fig. 1 (see section on supplementary materials given at the end of this article) (~87% knockdown in subcutaneous, ~73% in epidydimal and ~50% in mesenteric adipose tissues). Mice lacking *H6pdh* (generated using homologous recombination in embryonic stem cells to replace exons 2 and 3 with a neomycin resistance cassette (Lavery *et al.* 2006, 2008)) and their wild-type controls on a C57BL/6J background were generated in the University of Birmingham by heterozygous breeding and transferred to Edinburgh at the age of 3 months, under supervision of the Named Veterinary Surgeons at the Universities of Birmingham and Edinburgh. C57Bl/6J mice (Harlan Olac, Bicester, UK) were used to assess parameters for infusion to achieve steady state. Weights of animals are given in Supplementary Table 1. Studies were refined to be conducted in male mice only to permit the comparison of multiple genotypes within practical experimental constraints.

### *In vivo* experimental protocols

All experiments on animals were carried out in accordance with the UK Home Office Animals (Scientific Procedures) Act of 1986 and European Directive 2010/63/EU, following approval by the University of Edinburgh Animal Welfare and Ethical Review Body and the Named Veterinary Surgeon. LKO and AKO genotypes were assigned by *Cre* positivity/negativity as described (Zou *et al.* 2018) and by PCR of tail biopsy as described previously in mice lacking *H6pdh* (Lavery *et al.* 2006).

Mice were group housed (2–5 per cage) under controlled conditions: 12 h light/darkness cycle at 21ºC with free access to standard rodent chow and water. Except for *H6pdh^−/−^* mice and their controls, mice were housed in standard cages. *H6pdh^−/−^* mice and their controls were housed in independently ventilated isolators for 1 week prior to experimentation due to transfer between facilities. Mice (*n* = 3–6/group; exact group sizes indicated within legends) were infused with d4F (1.75 mg/day), at a rate of 1.03 µL/h by sub-cutaneous osmotic mini-pumps (ALZET model 1003D or 1007D; Cupertino, CA, USA; vehicle dimethylsulfoxide: propylene glycol (50:50)), primed as per manufacturer’s instructions and surgically implanted dorsally under isoflurane anaesthesia with veterinary-approved aseptic technique. Buprenorphine analgesia was administered peri-operatively, and mice were allowed to recover in individual warm boxes for ~60 min before being returned to their home cages in groups. Post-operative welfare-related assessments were carried out under veterinary guidance for the duration of the infusion period. To assess timing to achieve steady state C57Bl/6J mice were euthanised by decapitation after infusion intervals of 24 h, 48 h or 7 days, with a further group receiving vehicle until 7 days. Genetically modified lines were similarly culled for comparison with their respective controls after infusion for 48 h. Plasma was prepared from trunk blood (collected in EDTA-coated tubes) and tissues (liver, brain and adipose tissue) snap-frozen in liquid nitrogen and stored at −80°C.

### Quantitation of plasma and tissue steroids

The analyst was blinded to genotype. Steroids were quantified in plasma by liquid chromatography tandem mass spectrometry (LC-MS/MS) as described by Cobice *et al.* (Cobice *et al.* 2017). Tissue steroids were assessed by MS using two analytical approaches, matrix-assisted laser desorption ionisation (MALDI-MS) and LC-MS/MS. Methods previously reported for analysis of tissue steroids by Cobice *et al.* (Cobice *et al.* 2017) were refined to reduce ion suppression, improve the limit of quantitation and thus allow detection of tracers in adipose tissue.

#### Analysis of corticosteroids in tissue extracts by LC-MS/MS

Snap-frozen adipose tissue (50–70 mg) was homogenised in acetonitrile + 0.1% formic acid (500 μL) using a bead homogeniser (Bead Ruptor Elite, Omni International, USA, 1 cycle of 20 s at 4 m/s maximum speed; Kennesaw, GA, USA) and enriched with internal standards (1 ng; d8E and d8B). A calibration curve of analytes was prepared alongside the samples covering amounts in the range 0.0025–10 ng. Samples were subject to centrifugation (6000 ***g***, 5 min, 4°C) and the supernatant (500 μL) was spun down through a centrifugal tube filter (0.22 µm SpinX Nylon; 13,000 ***g***, 10 min, 4°C). Samples and standards were transferred to an ISOLUTE^®^ PLD+ 96-well plate cartridge (Biotage, Uppsala, Sweden), elution was performed under positive pressure (~15 psi, 10 min) and the eluate was reduced to dryness under oxygen-free nitrogen (OFN, 40°C). The samples were dissolved in mobile phase (water:methanol, 70:30; 100 μL) shaken for 5–10 min on an orbital plate shaker (250 rpm) and sealed before LC-MS/MS analysis using a Nexera 2 MP uHPLC system (Shimadzu, Kyoto, Japan) coupled to a QTRAP® 6500+ (SCIEX, Warrington, UK) equipped with a Turbospray interface in electrospray ionisation mode and operated with Analyst software v1.6.3. Separation was achieved on a Kinetex^®^ C18 column (150 × 2.1 mm, 1.7 μm, Phenomenex, Macclesfield, UK). Mobile phase (water (A) and methanol (B) each containing 0.05 mM NH_4_F) was used at a flow rate of 0.3 mL/min, with an initial hold of 3 min at 50% B followed by a linear gradient to 100% B over 10 min and then re-equilibration to initial conditions (2 min). Column and auto-sampler temperatures were 50°C and 10°C, respectively. Data were acquired by multiple reaction monitoring (collision energy, declustering potential, cell exit potential) of protonated molecular ions: d4F *m*/*z* 367→121 (29, 80, 16 V); d3F *m*/*z* 366→121 (25, 121, 20 V); d3E *m*/*z* 364→164 (31, 166, 14 V); d8E *m*/*z* 369→169 (33, 96, 20 V); corticosterone *m*/*z* 347→121 (29, 76, 8 V) and d8B *m*/*z* 355→125 (31, 56, 8 V) with retention times of 2.8, 2.85 and 2.9 min for d3F, d4F and F, 3.4 and 3.5 min for d8E and d3E and 5.3 and 5.35 min for d8B and corticosterone, respectively. Peak area ratios of steroids to corresponding isotopically labelled internal standard analogues were calculated and compared with corresponding calibration standards to generate a ng/g of tissue value.

### Analysis of corticosteroids in tissue extracts by MALDI-MS

Liver (~60 mg), adipose tissue (~100 mg) or half brain (sagittal, without pituitary, ~200 mg) were mechanically homogenised in methanol:water (3 mL; 70:20 v/v) and were enriched with internal standard, d8B (10 ng). Homogenates were shaken (15 min, RT) followed by centrifugation (4000 ***g***, 45 min, 4°C). Supernatants of liver and adipose tissue and half of the brain extract were reduced to dryness under OFN (RT; 40°C) in Reacti-vials. Residues were reconstituted in Girard T reagent (GirT; 100 μL, 5 mg/mL in methanol with 0.2% v/v trifluoroacetic acid) and incubated (40°C, 60 min) and allowed to cool to RT and reduced to dryness under OFN. For MALDI-FT-ICR-MS analysis, CHCA (200 μL, 10 mg/mL in 6:4 acetonitrile:water + 0.2% v/v TFA) was added, vortexed and spotted (1 μL) on a MALDI stainless steel plate, allowed to dry and stored under vacuum until analysis.

Tracer enrichment was assessed using a 12T SolariX MALDI-Fourier transform ion cyclotron resonance-MS (Bruker Daltonics, Billerica, MA, USA) employing a Smart beam 1 kHz laser and operated with SolariX control v1.5.0 (build 42.8). All analyses were carried out using 1000 laser shots and 1000 Hz laser frequency in positive ion mode. Laser power was optimised at the outset and then fixed for all experiments. Laser focus was set to a minimum (99.7%) and 150 μm areas of the spotted sample (1 μL) on Bruker MTP targets steel plate were ionised by a random walk. An average of 10 spectra within *m/z* 150–3000 was acquired using continuous accumulation of selected ions (CASI^TM^) mode to increase the signal-to-noise of the ions with an isolation window at *m/z* 458 ± 50 Da and collected with a 4 Mword time-domain transient to resolve all peaks. The GirT derivatives of d4F (d4F-GirT), d3F (d3F-GirT) and d3E (d3E-GirT), corticosterone-GirT and d8B (d8B-GirT), were monitored at *m/z*, 480.3370, 479.3307, 477.3151, 460.3166 and 468.3672, respectively. Spectral characterisation of steroids was assessed using a standard mix (100 pg, methanol:water (1:1)) of d4F and d3F spotted onto the same MALDI plate. Accurate masses were aligned with individual spectra (Bruker Compass Data Analysis software v4.1), and a single-point calibration against the abundance of d8B-GirT ion was applied.

### Data and statistical analysis

For tissue homogenates, the average intensities of the d4F-GirT, d3E-GirT, d3F-GirT and d8B-GirT ions are presented as ratios of derivatives of d4F/d8B, d3E/d8B, d3F/d8B, d4F/d3F and corticosterone/d8B. The GirT derivatives of labelled cortisol and cortisone yield very similar intensities upon quantitation by MALDI, and thus the ratio of abundances of the derivatives of the tracer steroids to internal standard was used for relative quantitation. The amount of internal standard (d8B) was normalised per milligram tissue. Concentrations of steroids measured by LC-MS/MS were quantified against linear calibration curves of peak area ratios of analytes versus internal standards, prepared concomitantly and accepted with a regression coefficient, *r* > 0.99. For quadrupole analyses, the intensities of deuterated steroids were corrected for the contributions of isotopologues with naturally occurring ^13^C and deuterium and assessed using reference standards; these species could be distinguished spectrally using FT-ICR-MS. Amounts of steroids in tissues were expressed as a ratio to the internal standard and corrected to 100 mg tissue.

Unless otherwise stated, data are expressed as mean ± s.e. of the mean and differences were analysed using a one-way ANOVA with Fisher’s post-test, Kruskal–Wallis with mean rank tests or Mann–Whitney U as appropriate (Statistica, Tibco, Palo Alto, CA, USA). Statistical significance was set at *P* < 0.05. Breeding of LKO and AKO mice was planned to achieve group sizes of a minimum of *n* = 5, allowing assessment of a 50% change of 11βHSD1 activity measured by d4F/d3F ratio to be detected in brain with a power of 90% (*P* = 0.05) (Cobice *et al.* 2017). The effect of disruption of *H6pdh* was assessed in groups of *n* = 3.

## Results

### Tracer turnover in tissues: timing of measurements

**Figure 1:**
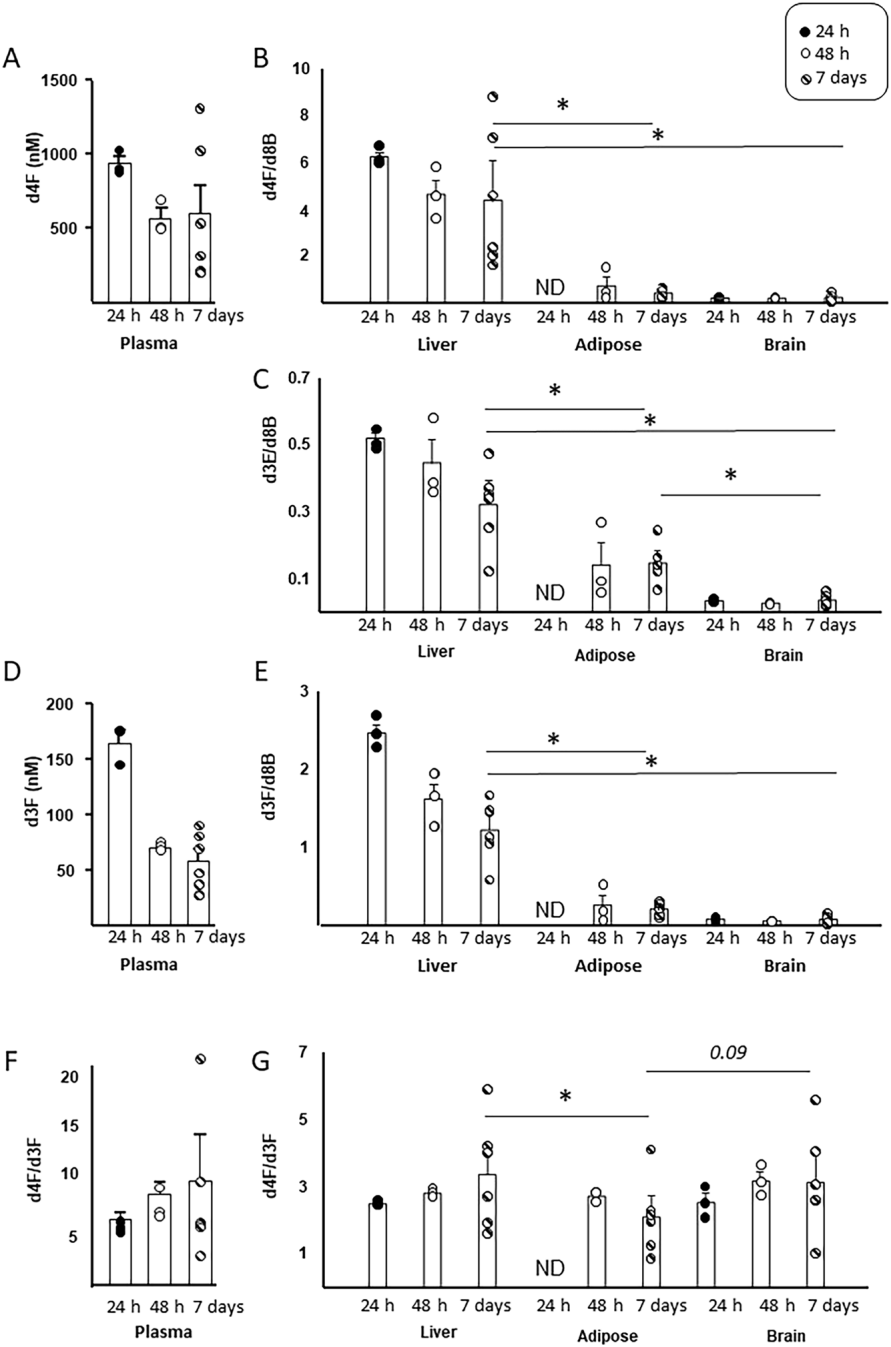
Tracer kinetics after timed infusions in mice. 9,11,12,12-[^2^H_4_]-Cortisol (d4F) was infused for 24 h, 48 h or 7 days into male C56BL6J mice (*n* = 3–6) by minipump. Steroids were quantified in blood (absolute concentrations) and tissues (liver, brain, gonadal adipose tissue; ratios vs d8-corticosterone (d8B; internal standard), respectively; d4F (A) and (B), d3E (C), d3F (D) and (E). D4F/d3F ratios are presented in plasma (F) and tissues (G). Individual data points are shown with mean and s.e. of the mean superimposed and compared in plasma by Mann–Whitney *U* tests and in tissues by one-way ANOVA (with Fisher’s post hoc test) at matched timepoints. *P < 0.05. ND = not detected.

Following tracer infusions for 24 h, 48 h or 7 days, concentrations of tracers in plasma and amounts in tissues were compared (Fig. 1). Tracers were not detected in plasma or tissue of mice receiving vehicle infusion, providing reassurance of lack of interfering analytical signals. In plasma, the concentrations of d4F decreased between 24 h and 48 h and then the mean value stabilised but with more variability between animals at 7 days (Fig. 1A). Similar to plasma, d4F concentrations in all tissues were highest at 24 h, stabilising between 48 h and 7 days with greater inter-individual variability at 7 days than at earlier times (Fig. 1B). Of the tissues tested, the highest amounts of d4F were found in liver at all time points, being ~10-fold higher than in adipose tissue and ~12-fold higher than in brain after 7 days infusion. D4F was undetectable in adipose tissue at 24 h but became detectable at the later time points. Endogenous corticosterone was detectable in plasma of control mice but not following infusion. The amounts of corticosterone reduced to around 5% of control levels in liver and brain by the 24-h time point and was undetectable by 7 days. In the case of adipose tissue, corticosterone abundance declined on average to ~51% (range 44–70%) of control amounts by 24 h, 31% (range 15–44%) by 48 h and remained around this level until 7 days (range 18–55%).

D3E was detected but could not be quantified in plasma due to the lack of a commercial analytical standard. Similar to d4F, d3F concentrations declined in plasma after 24 h, remained stable between 48 h and 7 days and showed greater inter-individual variability at 7 days than at earlier times (Fig. 1D). D3E and d3F were detected in liver and brain at 24 h but were undetectable in adipose tissue at this time point (Figs 1C and E). Amounts of both d3E and d3F were higher in liver than in other tissues and reduced after 24 h. Levels of both d3E and d3F remained stable in the adipose tissue and brain from 48 h to 7 days and their abundances in adipose tissue ultimately exceeded those of brain by 7 days.

Turnover of d4F to d3F as an indicator of 11βHSD1 activity was assessed as the d4F/d3F ratio the value of which is reduced with greater 11-keto reduction. The mean value of d4F/d3F ratio remained relatively stable during the 48 h to 7 day period in plasma, but again there was more variability between animals at the later time (Fig. 1F). The d4F/d3F ratio was lower in all tissues than in plasma at all time points and was similar in the brain and liver (Fig. 1G). The d4F/d3F ratio took longer to equilibrate in adipose tissue, achieving a lower ratio than in other tissues by day 7, but again was variable at this time point. Due to lower inter-individual variability, and having reached steady-state, 48 h was chosen as the most robust timepoint to compare mice of differing genotypes. In all subsequent experiments, findings in respective control mice were similar to those in the C57BL/6J mice.

### Influence of H6PDH on regeneration of D3F from D3E

**Figure 2:**
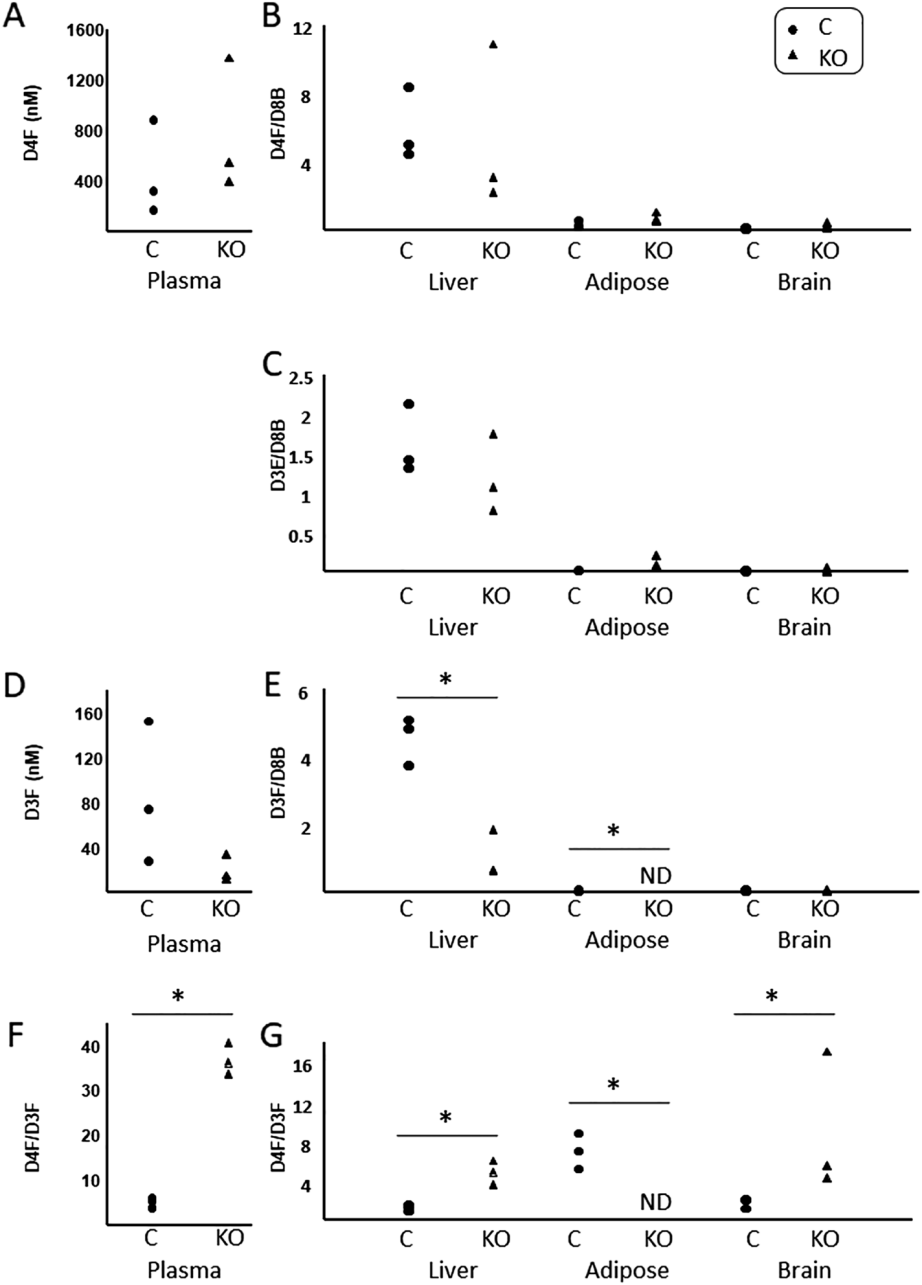
Tracer kinetics after timed infusions in mice with whole-body disruption of hexose-6-phosphate dehydrogenase. 9,11,12,12-[^2^H_4_]-Cortisol (d4F) was infused by minipump for 48 h into male mice (*n* = 3/genotype) with whole-body disruption of hexose-6-phosphate dehydrogenase (KO) and their littermate controls (C). Steroids were quantified in blood (absolute concentrations) and tissues (liver, brain, gonadal adipose tissue; ratios vs d8-corticosterone (d8B; internal standard) respectively; d4F (A) and (B), d3E (C), d3F (D) and (E). d4F/d3F ratios are presented in plasma (F) and tissues (G). Individual data points were compared in plasma by Mann–Whitney *U* tests and in tissues by Kruskal–Wallis ANOVA (with mean rank post hoc test). *P < 0.05. ND = not detected.

**Figure 3:**
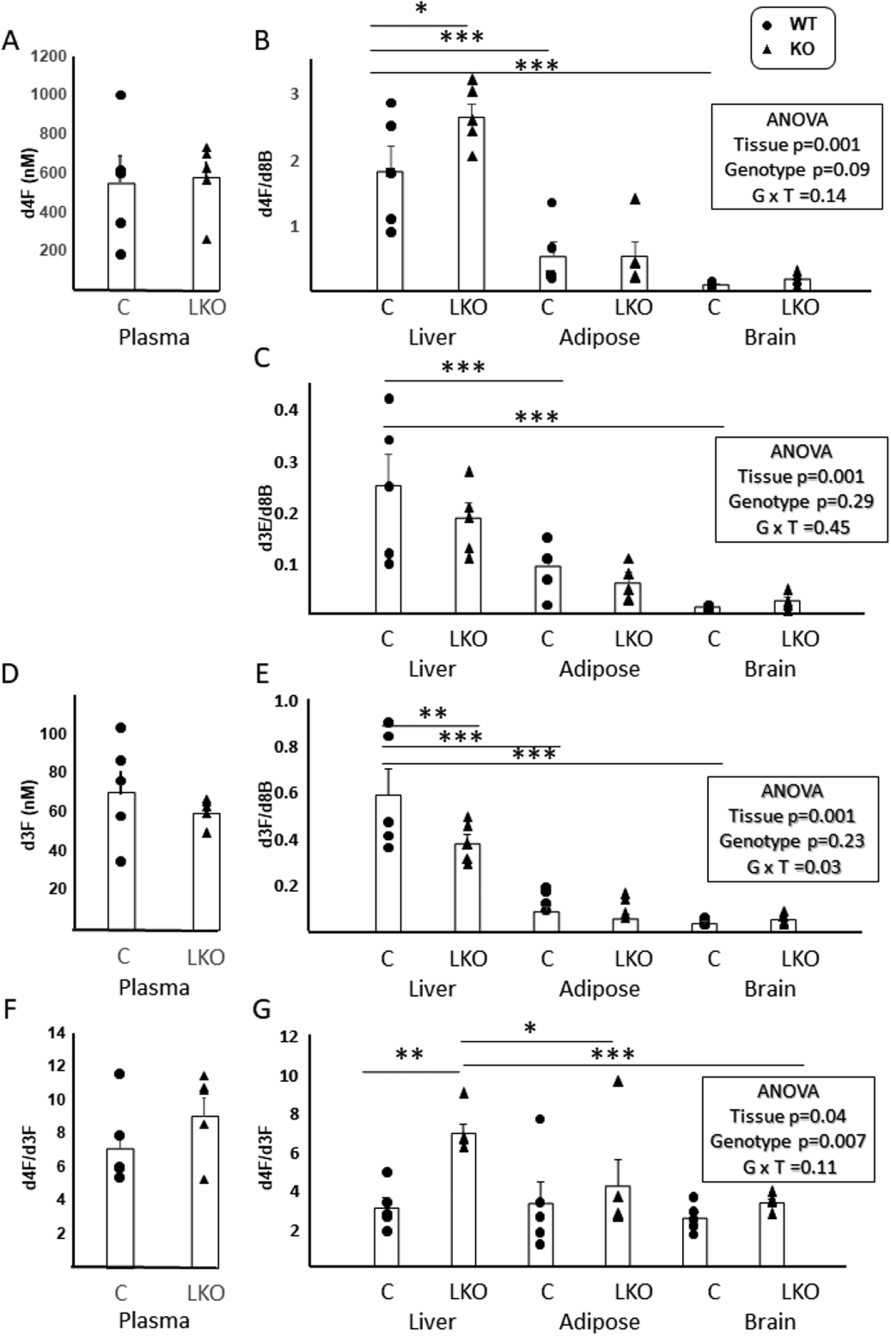
Tracer kinetics after timed infusions in mice with hepatocyte-specific disruption of 11β-hydroxysteroid dehydrogenase type 1. 9,11,12,12-[^2^H_4_]-Cortisol (d4F) was infused for 48 h into male mice with hepatocyte-specific disruption of 11β-hydroxysteroid dehydrogenase type 1 (LKO; *n* = 5) and their floxed littermate controls (C; *n* = 5) by minipump. Steroids were quantified in blood (absolute concentrations) and tissues (liver, brain, gonadal adipose tissue; ratios vs d8-corticosterone (d8B; internal standard), respectively; d4F (A) and (B), d3E (C), d3F (D) and (E). D4F/d3F ratios are presented in plasma (F) and tissues (G). Individual data points are shown with mean and s.e. of the mean superimposed and compared in plasma by Student’s *t*-tests and in tissues by two-way ANOVA (with Fisher’s post hoc tests). *P < 0.05, **P < 0.01***, *P* < 0.001. G = genotype, T = tissue.

**Figure 4:**
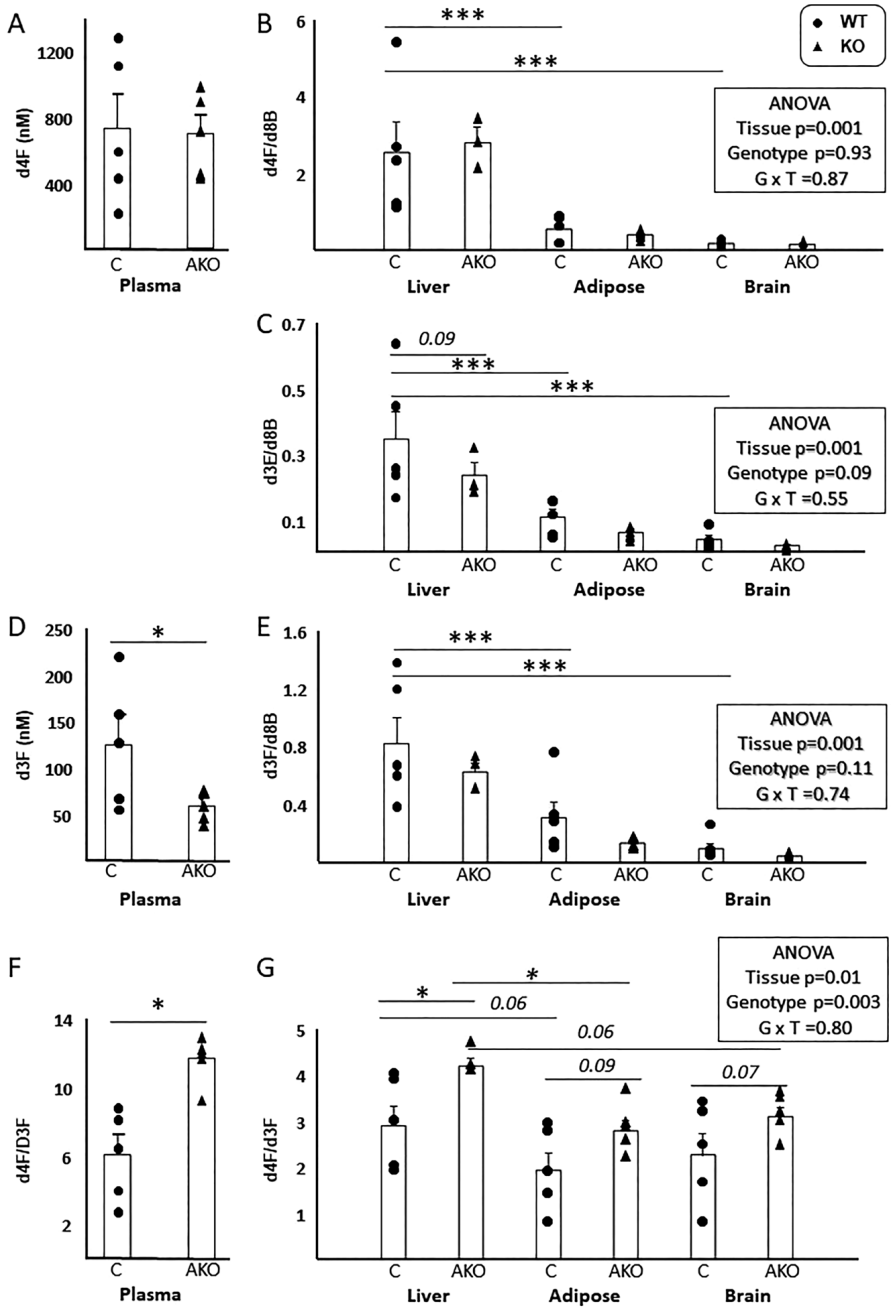
Tracer linetics after timed infusions in mice with adipocyte-specific disruption of 11β-hydroxysteroid dehydrogenase type 1. 9,11,12,12-[^2^H_4_]-Cortisol (d4F) was infused for 48 h into male mice with adipocyte-specific disruption of 11β-hydroxysteroid dehydrogenase type 1 (AKO, *n* = 5) and their floxed littermate controls (C, *n* = 5) by minipump. Steroids were quantified in blood (absolute concentrations) and tissues (liver, brain, gonadal adipose tissue; ratios vs d8-corticosterone (d8B; internal standard) respectively; d4F (A) and (B), d3E (C), d3F (D) and (E). D4F/d3F ratios are presented in plasma (F) and tissues (G). Individual data points are shown with mean and s.e.of the mean superimposed and compared in plasma by Student’s *t*-tests and in tissues by two-way ANOVA (with Fisher’s *post hoc* tests). *P < 0.05, ***P < 0.001. Trends are indicated if *P* < 0.1.

Given that H6PDH is important for 11βHSD1 reductase activity and thus generation of d3F from d4F (via d3E), we asked whether *H6pdh*^−/−^ mice could generate d3F to any degree and if so, were all tissues influenced similarly. Data are shown in Fig. 2. D4F was detected in similar concentrations in plasma of *H6pdh* ^−/−^ mice and their littermate controls (Fig. 2A). Regenerated d3F was detected in the circulation of both genotypes (Fig. 2D), but the d4F/d3F ratio was approximately six-fold higher in the plasma of *H6pdh*^−/−^ mice compared to their controls (Fig. 2F). This extrapolated into whole-body rates of appearance of d3F that were approximately six-fold lower in *H6pdh*^−/−^ mice than control, 0.05 ± 0.002 vs 0.33 ± 0.039 mg/day, respectively. These findings are consistent with 11βHSD1 reductase activity (driven by H6PDH) as a major but not exclusive contributor to plasma and tissue glucocorticoid regeneration. Moreover, the d4F/d3F ratio in the plasma of *H6pdh*^−/−^ mice was higher than in either of the mouse lines with tissue-specific disruption of 11βHSD1, in hepatocytes (Fig. 3F) or adipose tissue (Fig. 4F). Similarly in liver, d3F levels were lower in *H6pdh*^−/−^ mice, compared with controls (Fig. 2E) and the d4F/d3F ratio was higher overall in liver and brain in *H6pdh* ^−/−^ mice compared to controls (Fig. 3G). It was not possible to calculate a ratio for adipose tissue because d3F was not detected in that tissue in *H6pdh*^−/−^ mice.

### Steroid turnover in plasma and tissues in mice with hepatocyte-specific disruption of 11βHSD1

To investigate how hepatic 11βHSD1 contributes to the amounts of regenerated active steroid in the circulating pool as well as in liver, brain and adipose tissue, d3F was measured, comparing LKO mice to their respective controls following d4F infusion. Data are shown in Fig. 3. The concentrations of d4F and d3F in the circulation of LKO mice were unchanged compared with their littermate wild-type controls (Figs 3A and D), and consequently the d4F/d3F ratio was unchanged (Fig. 3F). These data equate to whole-body rates of appearance of d3F of 0.20 ± 0.03 vs 0.26 ± 0.03 mg/day in LKO vs control, respectively. Amounts of d4F were higher (Fig. 3B) and those of d3F lower (Fig. 3E) in liver of LKO mice compared to control littermates, without differences in adipose tissue and brain. In contrast to plasma, within the liver of in LKO mice the d4F/d3F ratio was higher (Fig. 3G) compared to littermate controls, again without any change in adipose tissue or brain. These data suggest that 11βHSD1 in liver makes a negligible contribution to circulating levels of active glucocorticoids in mice but is important for the intra-hepatic balance of active and inert glucocorticoids.

### Steroid turnover in plasma and tissues in mice with disruption of 11β-HSD1 in the adipose tissue

Similarly the contribution of 11βHSD1 in adipose tissue to circulating and tissue pools of regenerated glucocorticoids was studied, comparing AKO mice to their respective controls. Data are shown in Fig. 4. The concentrations of d4F in the circulation of AKO mice were unchanged compared with littermate controls (Fig. 4A), whereas those of d3F were lower in AKO mice (Fig. 4D). Accordingly, the ratio of d4F/d3F in plasma was higher in AKO mice than in littermate controls (Fig. 4F), revealing lower whole body rates of appearance of d3F in AKO mice (0.15 ± 0.02 vs 0.35 ± 0.08 mg/day; AKO vs WT, respectively). Moreover, the rate of appearance of d3F in plasma in the AKO mice was lower than that in LKO mice, but remained higher than in *H6pdh* ^−/−^ mice (each Mann−Whitney *U* test, *P* < 0.05). The d4F/d3F ratio was higher in all tissues of the AKO mice compared with their littermate controls (Fig. 4G).

## Discussion and conclusions

Hepatocyte-mediated glucocorticoid regeneration *in vivo* in male mice affected exposure to regenerated steroids only within the liver and not the circulation or measured tissues (adipose tissue or brain). This was not anticipated given that studies in humans show comparable rates of appearance of glucocorticoids across the liver to whole-body appearance measured in the circulation (Andrew *et al.* 2005, Basu *et al.* 2006, 2009). In contrast, disruption of 11βHSD1 expression in adipose tissue reduced levels of regenerated steroids in blood, liver and brain as well as in adipose tissue. Lastly it was shown that H6PDH plays a significant, but not exclusive, role in glucocorticoid regeneration in liver, brain and adipose tissue.

Overall, the regeneration of active glucocorticoid by 11βHSD1 measured using the d4F tracer in the mouse models was representative of previous results in humans, except steady state took longer to achieve in mice, around 48 h vs 3 h in humans, which may reflect differences between sub-cutaneous and intravenous infusion (Andrew *et al.* 2002). The temporal pattern of tracer appearance in the liver resembled that of plasma and in both cases the data became more variable between mice at 7 days. The reason for this was not investigated further but may have related to more variable delivery by the pumps towards the end of their operation, albeit they were designed to work for 1 week. The intermediate, d3E, and regenerated d3F stabilised by 48 h in blood and liver but at lower levels than d4F. The lower levels of d4F and d3F in brain compared with other tissues may reflect the active export of cortisol via the ABCB1 transporter. This transporter, which actively exports cortisol (Nixon *et al.* 2016) and, by inference, d4F and d3F, is expressed in brain. However, it is worth noting that tissues such as brain may have specific sub-regions of high expression of 11βHSD1, such as cerebellum and hippocampus (Moisan *et al.* 1990, Holmes *et al.* 2010), and greater regeneration of d3F in these regions may be diluted within whole tissue measures.

While fluctuations in circulating glucocorticoid levels are rapidly reflected in the hepatic pool of steroids, adipose tissue pool appears buffered in humans (Hughes *et al.* 2013) and responds in hours/days, reflecting longer-term, sustained changes in prevailing glucocorticoids. Plausibly this slow turnover might protect adipose tissue from intermittent and short-lasting surges of cortisol in the blood. Methodological improvements allowed the detection of tracer in adipose tissue, which had not been achieved in previous studies (Cobice *et al.* 2017) allowing the slower achievement of a steady state in adipose tissue than in brain or liver in mouse to be quantified. Endogenous corticosterone was also washed out of adipose tissue more slowly than other tissues. It is assumed that steroids enter cells by passive diffusion due to their lipophilic nature and are sequestered in lipid droplets in adipose tissue. The slow turnover of the adipose tissue pool, typical of lipophilic molecules (Bruno *et al.* 2021), may be due to the constrained efflux of steroids from the triglyceride-rich lipid droplets, whereas efflux from the more phospholipid-rich environment of the brain could be more rapid.

The contribution of 11βHSD1 in all tissues was evident through greater dilution of d4F with d3F (approximately three-fold) compared with plasma (measured as d4F/d3F ratio). Even in the absence of H6PDH, residual d3F generation could still be seen with lower d4F/d3F ratios in tissues than in blood. Previously, the d4F tracer had been administered to mice with global disruption of 11βHSD1 and d3F was not generated, indicative of 11βHSD1 being the sole enzymatic route of reduction of d3E to d3F. Here, the finding of small but residual regeneration of d3F in mice lacking H6PDH suggests that H6PDH is the main, but possibly not the sole, driver of 11β-reduction. The remaining 11β-reduction may be driven through an alternative source of co-factor, such as glucose-6-phosphate dehydrogenase (G6PDH); indeed patients with glycogen storage disease with perturbations in the G6PDH cycle do show disturbances in their HPA phenotypes (Rossi *et al.* 2020). The change in co-factor balance from predominantly NADPH towards NAPD in mice lacking H6PDH would be anticipated to alter the equilibrium of 11βHSD1 in favour of dehydrogenation (Lavery *et al.* 2006). This may have manifested in lower circulating concentrations of d4F under infusion conditions (as an indicator of increased clearance, including dehydrogenation). D4F concentrations in plasma were not significantly different in *H6pdh* ^−/−^ mice compared to their wild-type controls, however, this must be viewed with caution as group sizes were small and not designed to test such questions.

In mice with disruption of 11βHSD1 in either hepatocyte or adipose tissue, regeneration of d3F was attenuated in the tissue targeted by the genetic disruption, with altered 11-keto-reduction of tracer detected most sensitively through changes in the local d4F/d3F ratio. The circulating concentrations of d4F were not different between genotypes, and thus other clearance pathways, such as by 11βHSD2, appeared unaffected. The d4F/d3F ratio more than doubled within liver following hepatocyte disruption compared to around a 50% increase in the ratio in adipose tissue following adipocyte disruption in keeping with greater abundance of the enzyme in liver than adipose tissue (Chapman *et al.* 2013). The two-fold reduction in the amount of d3F in livers of the LKO mice compared to littermate controls suggests that under steady-state conditions roughly half of the active glucocorticoid within the liver derives from hepatic regeneration via 11βHSD1. The remaining d3F in liver is likely to be delivered from the blood, although it remains possible that non-hepatocyte cells may contribute to regeneration of hepatic d3F. Indeed Kupffer cells and vasculature both express 11βHSD1 and may contribute locally, though to a limited degree given >94% knockdown in hepatic *Hsd11b1* mRNA levels in LKO mice, compared to littermate controls (Zou *et al.* 2018).

It was striking that the increase in the ratio of d4F/d3F in liver was not evident in the circulation in LKO mice. It is possible that d3F regenerated from d3E within liver is routed through further irreversible metabolism, such as by A-ring reduction and conjugation, which might be studied in the future through urine or faecal collection. Alternatively, it may be actively transported into the bile rather than re-entering the circulation; glucocorticoids are excreted into the bile in rodents (albeit reports relate to endogenous corticosterone and not exogenous cortisol) (Morris 2015). Therefore, in rodents, cortisol regenerated by 11βHSD1 in liver may act in liver but not beyond, but it should be noted that the route of biliary excretion of glucocorticoids does not translate to humans (Morris 2015). These findings of restrained effects within the liver align with the subtle systemic metabolic phenotype of mice lacking 11βHSD1 in liver. However, it should be noted that these mice did have enlarged adrenal glands (Lavery *et al.* 2012), suggesting endocrine effects originating from the hepatic deficiency of 11βHSD1. Moreover, transgenic over-expression of 11βHSD1 in liver rescued the HPA axis phenotype of the global knockout mouse (Paterson *et al.* 2007). The HPA responses in this setting have been shown to be strain specific (Carter *et al.* 2009) thus this finding, and how it translates to humans, merits further exploration.

In contrast, adipocyte-specific disruption of 11βHSD1 changed the d4F/d3F ratio not only in adipose tissue but also in the blood, and remote tissues, liver and brain. This may be of pathological relevance in obesity which is associated with an increase in adipose tissue 11βHSD1 expression in humans (Rask *et al.* 2001, 2002), potentially modifying glucocorticoid action systemically. However, an important caveat is that *Ap2-Cre*, used to generate AKO mice, is expressed in cell types other than adipocytes, including macrophages, endothelial cells and certain regions of the brain (McInnes *et al.* 2012, Lee *et al.* 2013, Jeffery *et al.* 2014). Such ectopic expression may contribute to the phenotype, though this is unlikely to be of sufficient magnitude to explain the global effect observed (Lee *et al.* 2013). Moreover, Christy *et al.* showed that *Hsd11b1* was not expressed in the endothelial cells of blood vessels of male mice, being located instead in the smooth muscle (Christy *et al.* 2003).

The strengths of this study lie in the use of tracer kinetics which can distinguish sources of steroids, coupled with analysis by gold standard MS as opposed to less specific immunoassay which still dominates the preclinical literature. A unique set of genetic modifications have allowed dissection of the roles of the different tissues, with caveats discussed over the specificity of the *aP2-Cre*, which might be overcome by alternative Cre driver, e.g., adiponectin-Cre. Future opportunities exist using MALDI imaging to measure the distribution of the tracers within specific brain regions (Cobice *et al.* 2017). Only gonadal adipose tissues from young, male mice were studied with a view to comparing key tissues; however, the nature of different adipose tissue depots differ. Further studies in both sexes are required, particularly given the differences in the distribution of adipose tissue between sexes, as well as sexual dimorphism in enzyme activity (Jamieson *et al.* 2000) and other regulatory factors such as binding globulins (Toews *et al.* 2021) in rodents. It would also be valuable to study animals under dietary high-fat challenge or over-expressing *Hsd11b1* in metabolic tissues, e.g. simulating the up-regulation of enzyme expression in adipose tissue which occurs in human obesity in both sexes (Rask *et al.* 2001, 2002). Further glucocorticoid-target tissues may be of interest for similar studies including skeletal muscle and bone and investigation of whether kinetics of turnover change in pathophysiological states such as insulin resistance and obesity and models with genetic disruption of *Hsd11b1* in populations of brain cells would provide valuable insight. Lastly, care must be taken when extrapolating data to humans, although mouse models have proven helpful in the development of 11βHSD1 inhibitors.

In summary, the active glucocorticoid generated by hepatic 11βHSD1 is largely constrained to the liver in mice, whereas the steroid pool in adipose tissue undergoes slower turnover and drains active glucocorticoid into the circulation where it reaches other tissues through endocrine delivery. Thus, inhibitors which access 11βHSD1 in adipose tissue may have broader reaching effects compared with those just targeting the liver, including attenuation of glucocorticoid action in brain.

## Supplementary Materials

Supplementary Figure 1

## Declaration of interest

SPW, JRS and BRW are academic inventors on patents relating to 11β-hydroxysteroid dehydrogenase 1 inhibitors, owned by the University of Edinburgh and licensed to Actinogen Medical Ltd. SPW, JRS, RA and BRW are consultants to Actinogen Medical Ltd.

## Funding

The work was supported by British Heart Foundationhttp://dx.doi.org/10.13039/501100000274 (RG-05-00 8; 2011) and its Centre for Research Excellence (BHF RE/08/001; 2008-date), and the Wellcome Trust http://dx.doi.org/10.13039/100010269 (WT083184, 2008; 107049/Z/15/Z; 2016). BRW is a Wellcome Trust http://dx.doi.org/10.13039/100010269 Senior Investigator.
