# Supplementary figures and images for: Contribution of local regeneration of glucocorticoids to tissue steroid pools
Source: J Endocrinol. 2023 Jul 28;258(3):e230034. doi: 10.1530/JOE-23-0034 (PMC10448579; doi:10.1530/JOE-23-0034)

Khan et al Supplementary Figure 1

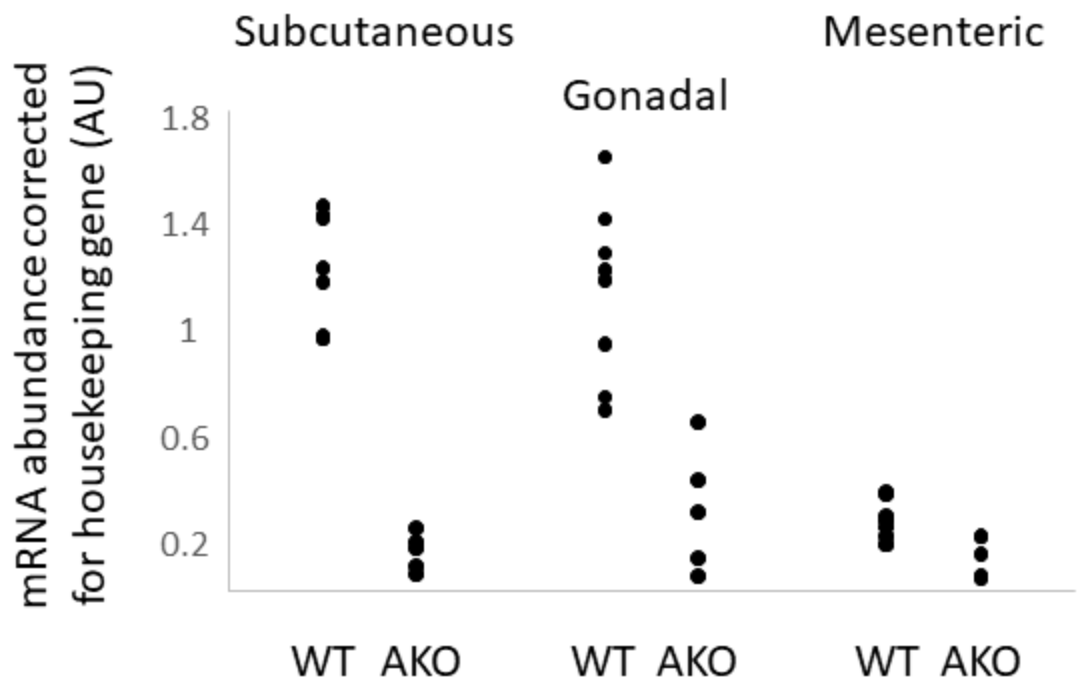

Supplement: Supplementary Figure 1 [file supplementary_figure_1.pdf]
